# Highly active selenium nanotherapeutics combined with metformin to achieve synergistic sensitizing effect on NK cells for osteosarcoma therapy

**DOI:** 10.1515/nanoph-2022-0289

**Published:** 2022-06-23

**Authors:** Yanxin Du, Zehang Zhang, Yu Yang, Ting Liu, Tianfeng Chen, Xiaoling Li

**Affiliations:** The Second Clinical Medical College, Guangzhou University of Chinese Medicine, Guangdong Provincial Hospital of Chinese Medicine, Guangzhou, China; Department of Oncology, Department of Chemistry, The First Affiliated Hospital, Jinan University, Guangzhou 510632, China; Institute of Food Safety and Nutrition, Jinan University, Guangzhou 510632, China

**Keywords:** immunotherapy, metabolism, natural killer cells, selenium nanoparticles, selenoprotein

## Abstract

NK cells-based cancer therapy combined with chemotherapeutic drugs for the treatment of tumors can enhance the immunosensitivity of NK cells, increase the expression of NK cell receptors, and eventually boost the killing effect of NK cells on cancer cells. Selenium (Se) with different chemical structures can be metabolized into selenoproteins to regulate tumor and immune cells’ fate and functions. Herein, we found that, functionalized Se nanoparticles (SeNPs) combining with metformin (met) could amply the immunotherapeutic effects of NK92 cells against osteosarcoma cancer. The results revealed that TW80-SeNPs combined with met had the optimum performance on NK92 cells for HepG2 cells, owing to the increased ROS in HepG2 cells and the augmented expression of cell surface receptor proteins ULBP-3/4, PD-L1, MICA, and NK92 cell surface receptor proteins PD-1 and FasL. Additionally, TW80-SeNPs were gradually metabolized into selenoproteins (Gpx4 and TR1) into human osteosarcoma MG63 cells to reinforce the anticancer effect of NK92 cells by regulating the redox balance in the tumor microenvironment. This study provides a therapeutic approach in treating cancer itself or diabetes coupled with cancer. Moreover, it provides a multidrug strategy to improve immune cell function in practical applications, especially for synergistic immunotherapy of osteosarcoma.

## 1 Introduction

Many studies have found that the longer the duration of the onset of diabetes, the greater risk of cancer morbidity, which is 2–3 times higher than the incidence of nondiabetic patients [1, 2]. Its possible carcinogenic mechanisms are the patient’s own hyperglycemia, own chronic inflammation, obesity, and hyperinsulinemia. Hyperglycemia can cause oxidative stress by glycosylation of hemoglobin to release ferrous ions (as a pro-oxidant) for generating free radicals [3, 4]. Autologous chronic inflammation induces expression of tumor necrosis factor (TNF-α) and interleukin-6 (IL-6) [5, 6]. In order to maintain a normal insulin level, pancreatic beta cells secrete more insulin, resulting in hyperinsulinemia and further affecting the expression of matrix proteins related to liver cell damage, inflammation, and fibrosis [7, 8]. These complex conditions reduce immune system function and greatly increase the incidence of cancer.

Metformin (met), as a first-line clinical drug for type 2 diabetes (T2DM), is a widely used hypoglycemic drug that significantly affects treating diabetes. In return, this inexpensive and safe drug, which causes few side effects, attracted great attention [9, 10]. In recent years, with more and more studies of met in cancer treatment, it was found that met has inhibitory effects on breast cancer, hepatocellular carcinoma (HCC), prostate cancer, colon cancer and lung cancer [11–13]. Based on the current treatment situation, for many patients, met alone has limited therapeutic effects [14]. Therefore, that is crucial to find therapies in combination with met and possibly identify signaling proteins that are sensitive to met to improve met therapy and research on the pathways related to the occurrence of cancer [15]. In addition, met also showed good application prospects in tumor immunotherapy. Evidence indicated that met could enhance the cytotoxic function of T lymphocytes [16–18]. While the research of met on the function and metabolism of NK cells is rare, the effect of met on NK cell function needs to be further studied [19, 20].

As another mode of adoptive cell therapy, NK cell therapy is distinct from the chimeric antigen receptor T cell therapy strategy. It can selectively kill cancer cells by releasing cytotoxicity, activating apoptosis of target cells, and the combined approach with antibodies, with good safety and major histocompatibility complex nonrestrictive and other advantages [21, 22]. However, the insufficient targeting activity of NK cells to tumor cells and the tumor immunosuppressive microenvironment limit the function and activity of NK cells [23]. NK cells combined with chemotherapy drugs for the treatment of tumors can enhance the immune sensitivity of NK cells, increase the expression of NK cell sensitivity receptors, and enhance the killing effect of NK cells on cancer cells [24, 25]. It was reported that carbotinib can synergize NK92 cells to produce a more significant killing effect on renal cancer, in which carbotinib increases the expression of EGFR and decreases the expression of PD-L1 in tumor cells, reversing immunosuppression [26]. Our previous studies have reported that selenium-containing ruthenium complex (RuSe) and small-molecule drugs (SeC) can synergically enhance the killing effect of NK cells. RuSe mainly induces DNA damage by affecting the ROS level of prostate cancer cells, and then sensitizes the immune killing effect of NK cells through TRAIL/TRAIL-R and Fas/FasL mediated signaling pathways [27, 28]. Therefore, the combination of chemotherapy drugs and NK cells to enhance the immunotherapy effect on cancer cells is a good therapeutic strategy.

**Scheme 1:**
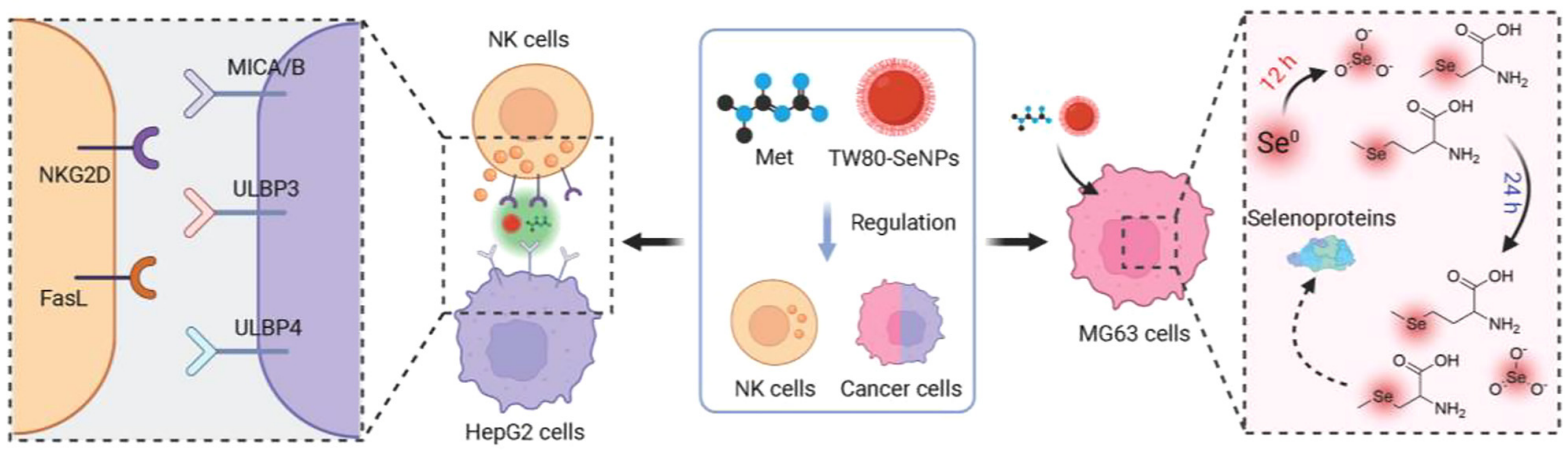
Schematic demonstration of TW80-SeNPs with met to achieve synergistic sensitizing effect on NK cells for cancer therapy.

The application of nanomaterials in biomedicine has received more and more attention [29–34]. SeNPs have the function of anti-oxidation and scavenging free radicals in normal cells, and can reduce the effect of oxidative stress caused by hyperglycemia [35–38]. On the other hand, SeNPs can inhibit cancer cells proliferation and regulate the function of immune cells to enhance the ability of immune cells to kill cancer cells [39, 40]. Moreover, Se plays an important role in bone-related diseases, including Kashin–Beck disease, Keshan disease, rheumatoid arthritis, osteosarcoma, and so on [41]. In addition, previous studies have found that functional SeNPs combined with met have synergistic anticancer effects [42]. Therefore, we further studied functional SeNPs combined with met to achieve a synergistic sensitizing effect on NK cells for cancer therapy (Scheme 1).

## 2 Materials and methods

### 2.1 Chemicals and materials

Acetic acid, perchloric acid, sodium chloride, and nitric acid were retrieved from the commercial companies located in China. DMEM medium were purchased from Invitrogen. Ascorbic acid (Vc), sodium selenite (Na_2_SeO_3_), poly (acrylamine hydrochloride) (PAH), polyvinylpyrrolidone (PVP), tween 80 (TW80) and the standards of selenium-based compounds including selenocystine (SeCys_2_), selenomethionine (SeMet), Na_2_SeO_3_ (SeIV), methylselenocysteine (MeSeCys), Na_2_SeO_4_ (SeVI) were retrieved from Sigma Aldrich. Moreover, cell lines such as hepatocellular carcinoma cells (HepG2) and human osteosarcoma cells (MG63) were retrieved from American Type Culture Collection (Manassas, VA).

### 2.2 Synthesis of selenium-based nanoparticles modified by different polymers

SeNPs were synthesized following the standard protocols. Briefly, reserve solution of 0.5 mL Na_2_SeO_3_ at the concentration of 100 mM was mixed with 1 mL of PVP, PAH, or TW80 water reserve solution at the concentration of 20 mg/mL, respectively. Thereafter, deionized water was added up to a total volume of 8 mL, followed by magnetic stirring for 5 min. Subsequently, the mixture was added with 2 mL of Vc reserve solution (100 mM) drop by drop and then stirred overnight at room temperature for 24 h. At last, the mixture solvents were dialyzed for 24 h to acquire functional SeNPs. Furthermore, the concentration of SeNPs was quantified against Se content via ICP-MS.

### 2.3 Cell survival rate assessment

The survival rate of cancer cells was determined according to the conventional MTT assay. HepG2 and MG-63 cells were inoculated in 96-well plates with a density of 2 × 10^4^ cells/mL for adherent growth 24 h. Then, functionalized SeNPs, NaSeO_3_, SeC, and met were added for 72 h of incubation. We also examined the cell viability of HepG2 and MG63 cells after treatment with SeNPs + met and SeNPs + met + NK92 (cancer cells: NK92 = 1:5) for 24 h.

Cell survival rates of NK92 cells were detected by CCK8 assay: NK92 cells were selected and inoculated into 96-well plates with a cell density of 1 × 10^5^ cells/mL or 2 × 10^5^ cells/mL. After co-incubation with the corresponding drug concentration for 48 h, the cell viability was performed by traditional cell counting kit-8 (CCK-8) assay and the 96-plates were read at 450 nm.

### 2.4 Cell surface receptor detected by flow cytometry

Cancer cells: Logarithmic HepG2 cells were selected and inoculated with an initial density of 1.0 × 10^5^ cells/mL. After 24 h, the corresponding drug concentration of TW80-SeNPs and met was added and incubated for 24 h. The cells were digested with trypsin, centrifuged, cleaned with PBS, and suspended again in PBS. The cells were suspended at 1.0 × 10^5^ cells/µL, and 1 µL of the corresponding antibody was added to every 100 µL of the cells, and the cells were incubated at room temperature under dark conditions for 1 h. After the incubation, the cells were washed, the supernatant was discarded and the corresponding secondary antibody was added for further incubation for 1 h. After incubation, the cells were washed, the supernatant was discarded, 200–400 µL PBS was added to resuspend the cells, and detection was performed by flow cytometry.

NK92 cells group: Logarithmic NK92 cells were taken and inoculated with a cell density of 1.0 × 10 ^5^ cells/mL, TW80-SeNPs and met with the corresponding concentration were added. After incubation for 24 h, the cells were resuspended by PBS after horizontal centrifugation at 1800 rpm for 5 min. The follow-up procedure was the same as that of the tumor group.

### 2.5 Examination of reactive oxygen species (ROS) in HepG2 cells

HepG2 cells were selected and inoculated with a cell density of 2.0 × 10^5^ cells/mL. After 24 h, corresponding concentration of TW80-SeNPs and met was added and incubated for 8 h. Then, the cells were collected for centrifugation and dyed with 500 nM DCF probe for 30 min. Finally, the cells were washed and resuspended in whit 300 µL PBS for examination by flow cytometry with channels of FSA, SSA, and FITC.

### 2.6 Cellular uptake of TW80-SeNPs in MG63 cells

MG63 cells were placed in a 6 cm dish at a density of 5 × 10^4^ cells/mL. After adding 40 µM coumarin-6-labeled TW80-SeNPs were incubated with the cells for different times. Then the medium was removed, and the cells were collected, washed with PBS, resuspended to 300 µL in PBS. Finally, the flow cytometry was used to measure the intracellular fluorescence intensity.

### 2.7 TW80-SeNPs metabolism in MG63 cells

The control group was not added with SeNPs or met, and the experimental group was treated with TW80-SeNPs with the final concentration of 50 μg/mL and 50 μg/mL TW80-SeNPs + 10 mM met for 12 h and 24 h. After 24 h, culture medium and tumor cell lysate were collected, respectively. Then HPLC-ICP-MS was used for analysis (Hamilton PRP X-100 Anion-Exchange Column). Standards for selenium-related metabolites (SeCys_2_, SeMet, SeIV, MeSeCys, and SeVI) were used for quantitative analysis of TW80-SeNPs metabolites.

### 2.8 X-ray photoelectron spectroscopy (XPS) of TW80-SeNPs in MG63 cells

After MG63 cells were adhered to the dish, TW80-SeNPs with a final concentration of 50 μg/mL and 50 μg/mL TW80-SeNPs + 10 mM met were added to the MG63 cells in the experimental group for 12 h, respectively. The upper medium was discarded, and the cells were cleaned twice with PBS. Then, the cells were gently scraped off with a cell scraper, centrifuged at 12,000 rpm for 10 min, and most of the upper PBS was removed, sealed in a liquid nitrogen tank for 2 min, followed by freeze-drying to obtain powder samples for XPS analysis.

### 2.9 Quantitative real-time polymerase chain reaction (qPCR)

Briefly, MG63 cells were treated with 20 μM TW80-SeNPs and 20 μM TW80-SeNPs + 10 mM met for 3 h and 6 h. Thereafter, MG63 cells were harvested and washed triplicate with pre-cooled PBS solutions. Total RNA of MG63 cells was isolated using Trizol solution and subsequently transcribed into cDNA with the aid of the PrimeScript™ RT reagent kit (Takara, Japan). Finally, qPCR assay was detected on CFX Connect™ real-time PCR detection system (Bio-rad, USA) using eight-strip PCR tubes at a final volume of 20 μL. The transcript abundance of related genes was examined by the 2^−ΔΔCt^ method normalized to that of the reference gene GAPDH.

### 2.10 Statistical analysis

All experiments were performed at least triplicates and the released data were shown as mean ± SD using SPSS 13.0 for statistical analysis. The differences between the control and treatment groups were analyzed using the two-tailed unpaired Student’s t-test at a statistical significance of *p* < 0.05 (^*^), *p* < 0.01 (^**^) or *p* < 0.001 (^***^).

## 3 Results and discussion

### 3.1 Functionalized SeNPs combined with met enhances the immunotherapy effect of NK92 cells for HCC

To investigate whether functionalized SeNPs combined with met can enhance the killing effect of NK-92 cells on cancer cells. Firstly, we examined the cytotoxicity of functionalized SeNPs and met on HepG2 cells. As shown in Figure S1A, the toxicity of PAH-SeNPs to HepG2 cells had a concentration gradient effect in the range of 5–80 µM, and the cell viability was 97%, 85%, 64%, 25%, and 16%, respectively, which indicated that PAH-SeNPs was low cytotoxicity at concentrations of 5 µM and 10 µM. However, the cell viability of PVP-SeNPs and TW80-SeNPs treated HepG2 cells presented a horizontal trend in the range of 5–80 µM, and the cell survival rate was stable in the range of 60–70%. In the range of 5–80 mM, met also showed a concentration gradient effect on HepG2 cells (Figure S1B). At 20 mM, 40 mM, and 80 mM, met had a survival rate of 61%, 15%, and 9%, respectively, showing high toxicity to cells. The cell survival rate at 5 mM and 10 mM was 95% and 81%, respectively, showing low toxicity to cells. Based on the above results, we selected concentration gradients with Se concentration of 2.5 and 5 µM and met concentration of 5 mM and 10 mM for subsequent experimental studies.

Secondly, we used CCK8 kit to examine the toxicity of SeNPs and met on NK92 cells. These results demonstrated that PAH-SeNPs and PVP-SeNPs showed certain toxic effects on NK92 cells at 20 and 40 µM Se concentrations in the range of 5–40 µM Se (Figure S2A and B). And no significant toxicity was observed at concentrations of 5 µM and 10 μM. TW80-SeNPs had no obvious toxicity to NK92 cells (Figure S2C). In the range of 5–40 mM met concentration, to some extent, met boosted the growth of NK cells (Figure S2D).

**Figure 1:**
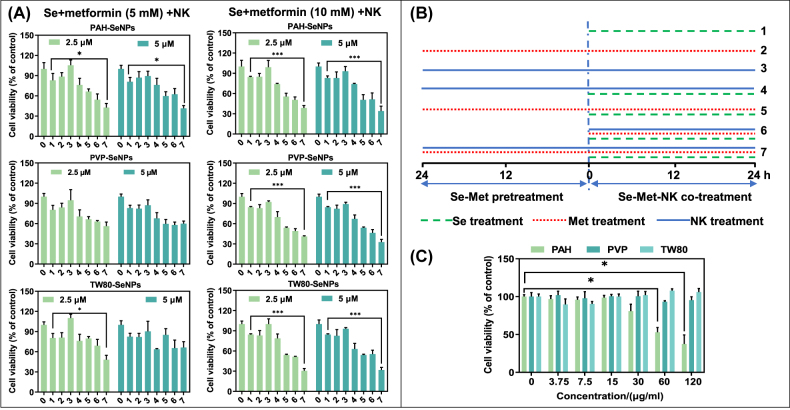
The cell viability of HepG2 cells after functionalized SeNPs combined with met and NK92 cells treatment. (A) The cell viability and (B) schematic diagram of different processing methods of the functionalized SeNPs combined with met and NK92 cells. **(C)** The cell viability of the polymer PAH, PVP and TW80 for HepG2 cells. Each value represents means ± SD (*n* = 3), ^*^ *p* ≤ 0.05, ^**^ *p* ≤ 0.01, ^***^ *p* ≤ 0.001.

Finally, we examined whether functionalized SeNPs combined with met could enhance the therapeutic effect of NK92 cells for HCC. As shown in Figure 1A and B, we found that HepG2 cells were pretreated with functionalized SeNPs combined with met (10 mM) and then co-treated with NK92 cells, which had an obvious killing effect on HepG2 cells. Moreover, sequence of enhancement effects were PAH-SeNPs > TW80-SeNPs > PVP-SeNPs. We also evaluated the toxicity of PAH, TW80 and PVP on HepG2 cells. As shown in Figure 1C, the 60 and 120 μg/mL of polymer PAH had certain toxicity to HepG2 cells corresponding to the Se concentration of 40 µM and 80 µM, while PVP and TW80 had no significant toxicity to HepG2 cells. Based on the above experimental results, we selected TW80-SeNPs combined with met (10 mM) to pretreat cancer cells for 24 h and then added NK92 cells as a follow-up experimental study of related anticancer mechanisms.

### 3.2 Enhancement of patient-derived NK cells by TW80-SeNPS combined with met

**Figure 2:**
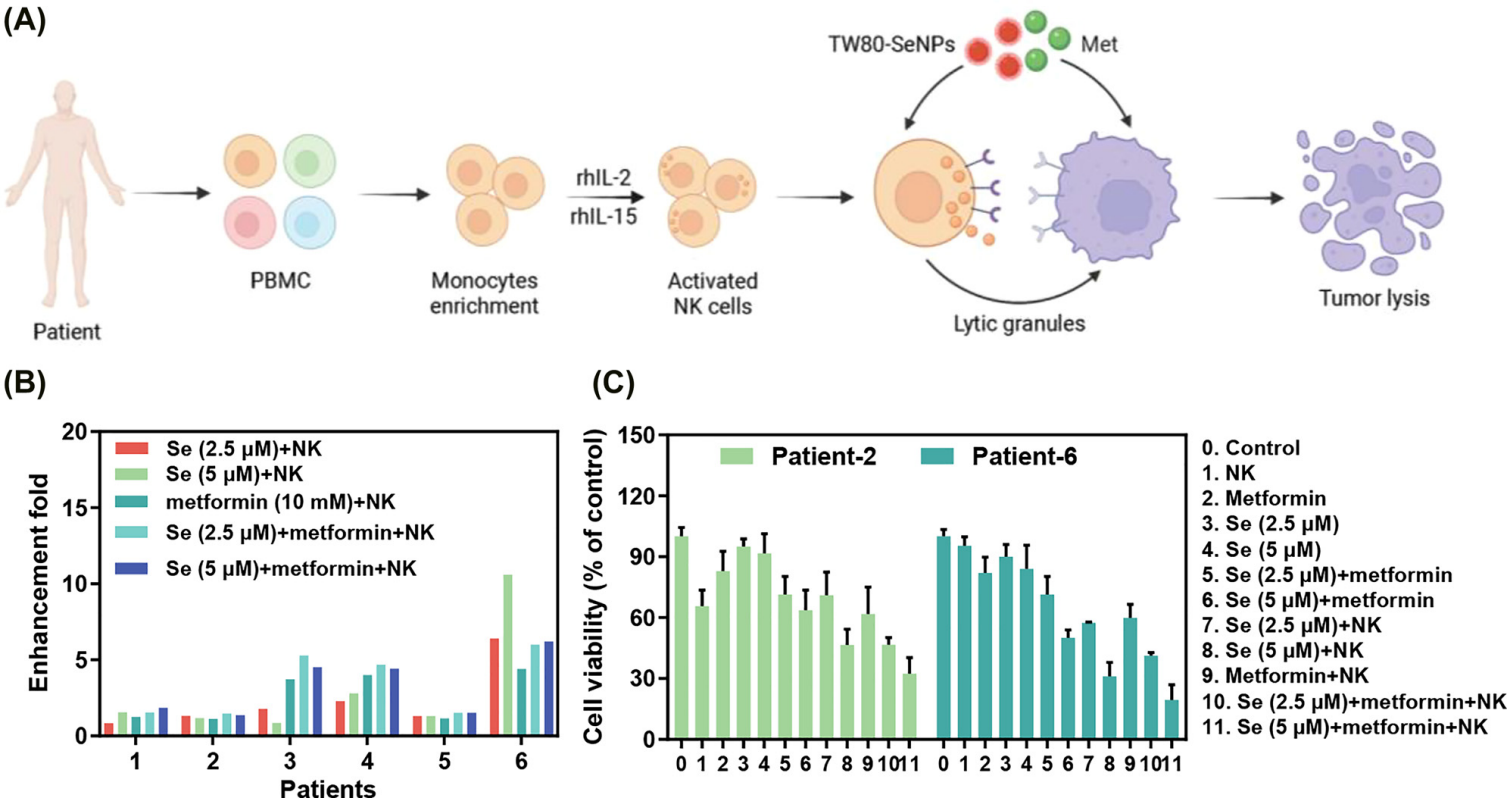
The enhancement fold changes of the combined treatment were calculated as the cancer cell growth inhibitory ratio percentage of NK cells alone. (A) The illustration of TW80-SeNPs + met enhances the anticancer effects of NK cells from blood of patients. (B) The enhancement fold and (C) the cell viability of patient-2 and patient-6 for HepG2 cells. Each value represents as means ± SD (*n* = 3).

TW80-SeNPs + met can enhance the immunotherapy effect of NK-92 cells. Can the combined treatment enhance the therapeutic effect of NK cells from patients? Therefore, we examined the immunotherapeutic effect of TW80-SeNPs + met on NK cells from 6 patients (Figure 2A). As shown in Figure 2B, the immunotherapy effect of TW80-SeNPs + met on NK cells of 6 patients was increased by 0.8–10 times, and there were some differences in the enhancement multiple of NK cells from different patients. The enhancement multiple of case 1 ranged from 0.8 to 1.9 times, that of case 2 ranged from 1.1 to 1.5, that of case 5 ranged from 1.1 to 1.5; while that of case 3 ranged from 0.8 to 5.2, that of case 4 ranged from 2.8 to 4.6 and that of case 6 was between 4.4 and 10.6 (Figure 2B). As shown in Figure 2C, the cell viability of TW80-SeNPs + met + NK group were 32% and 19%, respectively. There were significant differences compared with the NK (case 2 and case 6) group alone (65% and 95%) and TW80-SeNPs + met group (63% and 51%). Therefore, TW80-SeNPs + met can enhance the immunotherapy effect of NK cells in patients.

### 3.3 Regulation of TW80-SeNPs combined with met on receptor expression

**Figure 3:**
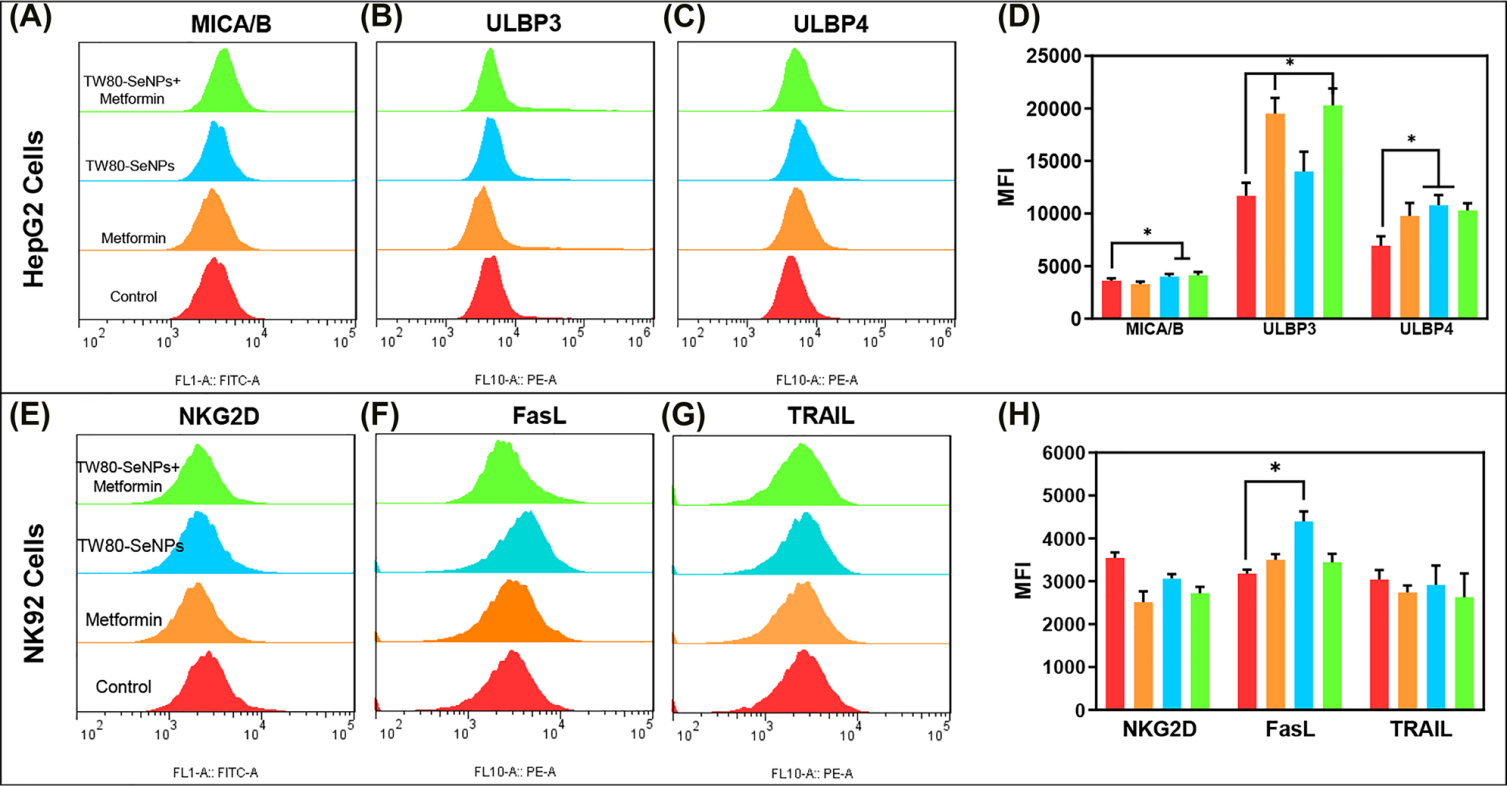
The expression levels of receptor signaling molecule on HepG2 cells and NK92 cells after treatment with TW80-SeNPs combined with met for 24 h. (A) MICA/B, (B) ULBP3 and (C) ULBP4, (D) the MFI value of each receptor signaling molecule on HepG2 cells, (E) NKG2D, (F) FasL and (G) TRAIL, and (H) the MFI value of each receptor signaling molecule on NK92 cells. Each value represents as means ± SD (*n* = 3), ^*^ *p* ≤ 0.05, ^**^ *p* ≤ 0.01, ^***^ *p* ≤ 0.001.

Soluble MHC class I molecule-associated protein (MICA/B), ULBPs family protein (ULBP-1/2/3/4) are functional ligands that bind to NK92 cell surface signaling receptor (NKG2D), and the increase of their expression can enhance the recognition of NK92 cells to cancer cells, thereby affecting the immunotherapy effect of NK92 cells [43]. Programmed cell death ligand (PD-L1) of cancer cells can bind to programmed death receptor 1 (PD-1) on the surface of immune cells to enhance immune evasion of cancer cells [44]. Therefore, we investigated the expression of signal receptors on HepG2 cell surface after treatment with TW80-SeNPs + met. As shown in Figure 3A and D and S3C and D, it was found that the expression level of MICA significantly increased in the Se group and the drug combination group, while the expression level of PD-L1 decreased slightly in the drug combination group. The expression level of ULBP-1/2 in the treatment group showed a downward trend or no significant change (Figure S3A, B and D), while the expression level of ULBP-3/4 was significantly increased in the treatment group (Figure 3B–D).

We also detected the expression levels of PD-1 and NKG2D in NK92 cells. As shown in Figure S3E and F, the expression level of PD-1 in the treatment group increased slightly, while the expression level of NKG2D decreased (Figure 3E and H). In addition, research reported that Se-containing compounds can induce the expression of death receptor-related proteins FasL and TRAIL on NK cells, and then kill cancer cells through the Fas/FasL and TRAIL/TRAIL-R pathways. Therefore, we also detected the expression of FasL and TRAIL in NK92 cells after treatment. From Figure 3E–H, we found that the expression level of FasL in the treatment group increased dramatically, while the expression level of TRAIL decreased changed slightly. According to the above result, we speculate that the increased expression of ULBP-3/4 and MICA signals in HepG2 cells and FasL signals in NK92 cells may be the main reason for enhancing the immunotherapy effect of NK92 cells.

### 3.4 TW80-SeNPs combined with met increased ROS level of HepG2 cells

In the process of immunotherapy, the increase of ROS in cancer cells induces the expression of corresponding signaling pathway proteins, thus enhancing the binding between immune cells and tumor cells, and playing an auxiliary role in enhancing the killing effect of immune cells on cancer cells [24, 45]. Therefore, we studied the changes of ROS levels in HepG2 cells after treatment with TW80-SeNPs and met for 8 h. After being treated with TW80-SeNPs and met alone, the intracellular ROS level can be significantly enhanced, and the combined treatment of TW80-SeNPs and met can further improve the intracellular ROS level (Figure S4). Therefore, the increase of ROS levels in cancer cells plays an important role in enhancing the immunotherapy of NK92 cells.

### 3.5 Functionalized SeNPs combined with met enhance the immunotherapy effect of NK92 cells for human osteosarcoma

**Figure 4:**
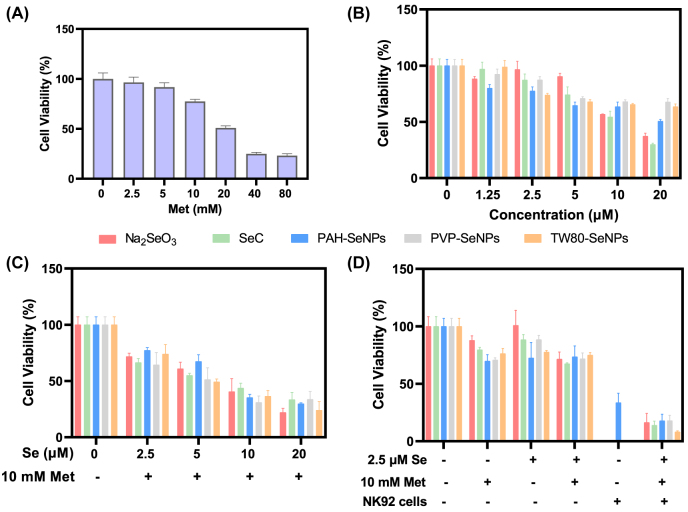
The cell viability of different SeNPs combined with met and NK92 cells in MG63 cells. The cell viability of (A) met, (B) different SeNPs, and (C) SeNPs combined with met in MG63 cells for 48 h. (D) The cell viability of different SeNPs combined with met for pretreatment 24 h and then NK92 cells co-treatment for 24 h in MG63 cells. Each value represents as means ± SD (*n* = 3).

Selenium plays an important role in osteogenesis and bone-related diseases [41, 46]. Among them, osteosarcoma is considered as the most regular bone cancer occurred in children and young adults. It is worthwhile noting that the improvement of survival rate of patients suffered from osteosarcoma remains challenging, although the breakthrough emerged in the therapies of this disease [47, 48]. Therefore, the cell viability of different SeNPs combined with met and NK92 cells in MG63 cells was determined by using human osteosarcoma MG63 cells. Figure 4A and B shows that the killing effect of different SeNPs or met on MG63 had a concentration gradient effect, and the combination of functionalized SeNPs and met have a synergistic killing effect on cancer cells (Figure 4C). Moreover, the combined killing effect of NK92 cells was further enhanced after administration for 24 h, while TW80-SeNPs showed the most significant synergistic enhancement (Figure 4D). According to the above results, TW80-SeNPs was selected for the research of the absorption, metabolism and transformation in MG63 cells.

### 3.6 The absorption, metabolism, and transformation of TW80-SeNPs in MG63 cells

**Figure 5:**
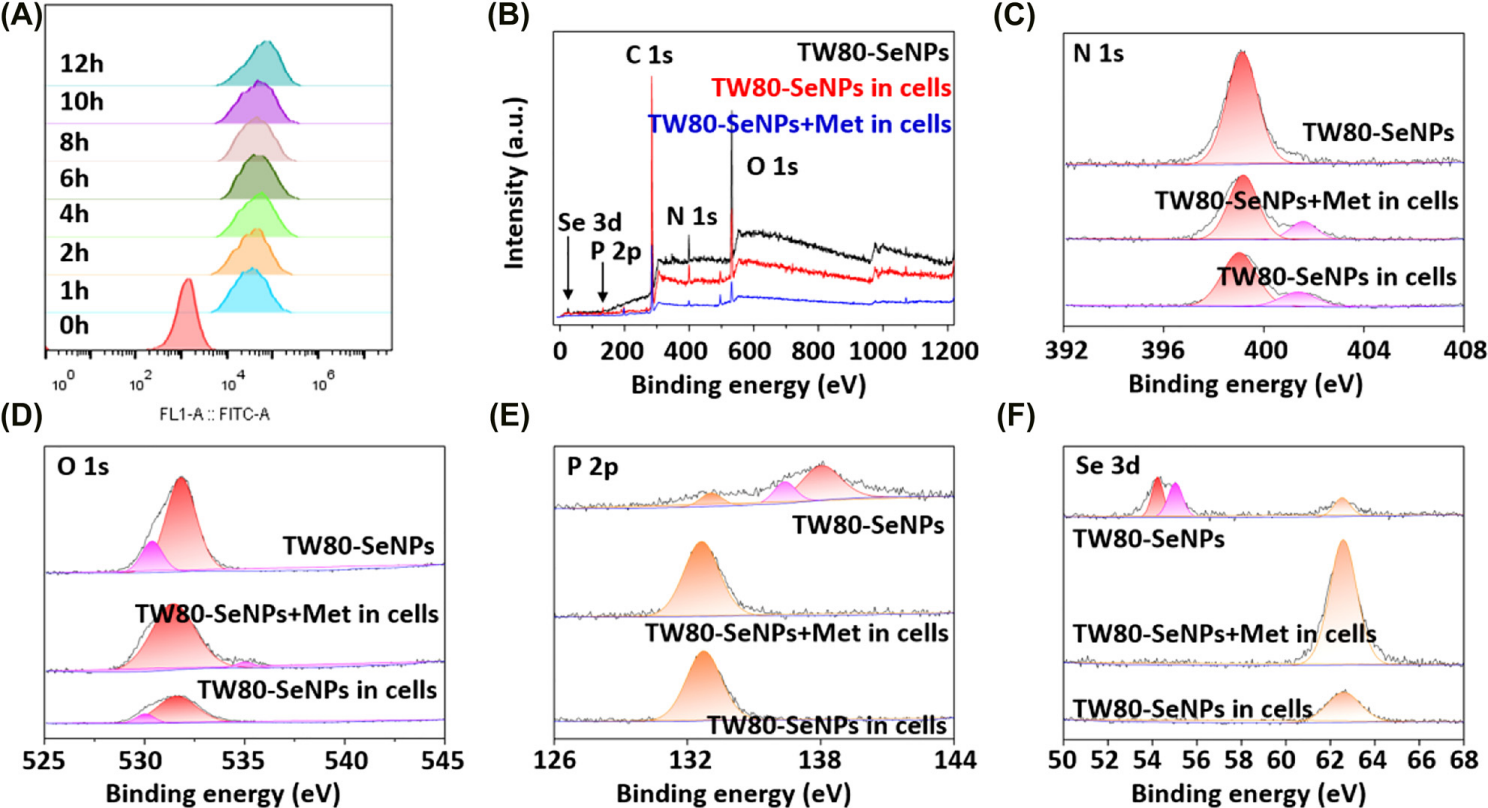
TW80-SeNPs absorption and transformation in MG63 cells. (A) Se uptake in MG63 cells. (B) XPS spectra of MG63 cells, (C) N 1s spectra, (D) O 1s spectra, (E) P 2p spectra, and (F) Se 3d spectra of MG63 cells after TW80-SeNPs and TW80-SeNPs + met treatment.

In order to fully understand how SeNPs play a role in cancer cells and explore the transformation process of selenium after entering cells, we measured the absorption of selenium by flow cytometry and XPS analysis. It can be seen from Figure 5A that the uptake of Se by MG63 cells does not increase significantly after 4–6 h, at which point Se has been basically absorbed by cells. Moreover, the XPS spectrum of Se samples presented featured bands corresponding to N, O, P, and Se shown in Figure 5B. And Figure 5C–F is N 1s Spectra, O 1s spectra, P 2p spectra, and Se 3d spectra of MG63 cells after treatment with TW80-SeNPs and TW80-SeNPs + met, respectively. It could be seen that there was a great difference in the spectra of TW80-SeNPs in MG63 cells compared to TW80-SeNPs. The TW80-SeNPs in cells group and TW80-SeNPs + met in cells group showed little difference in N 1s spectra and P 2p spectra, while the O 1s spectra and Se 3d spectra showed some difference, and the relative intensity was high after combined therapy.

**Figure 6:**
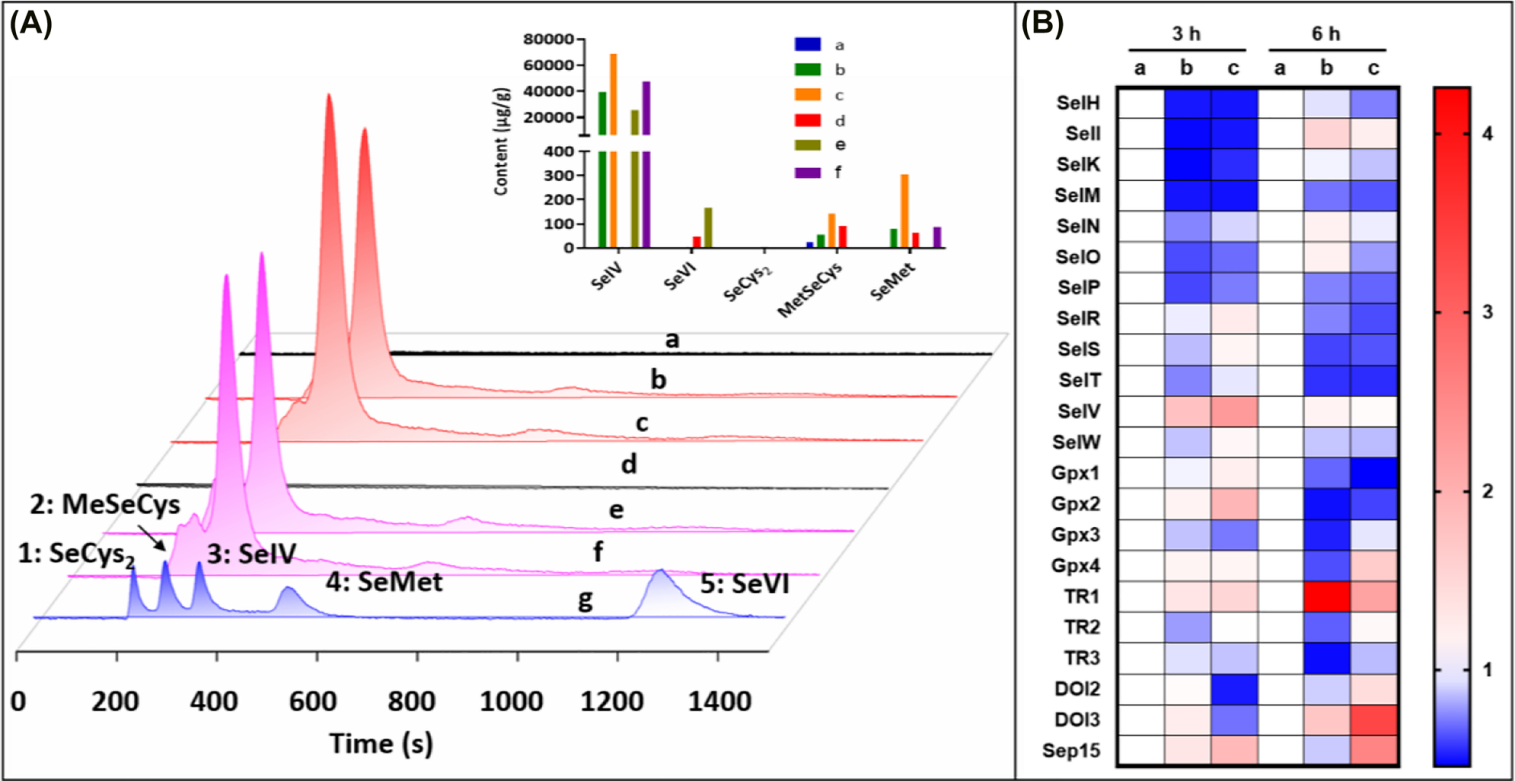
TW80-SeNPs metabolism in MG63 cells. (A) Se metabolites including SeIV, SeVI, SeCys_2_, MeSeCys, and SeMet in MG63 cells 12 h and 24 h after treatment using HPLC-ICP-MS analysis. The protein extraction solution of (a) MG63 cells for 12 h; (b) MG63 cells after TW80-SeNPs treatment for 12 h; (c) MG63 cells after TW80-SeNPs + met treatment for 12 h; (d) MG63 cells for 24 h; (e) MG63 cells after TW80-SeNPs treatment for 24 h; (f) MG63 cells after TW80-SeNPs + met treatment for 24 h. (g) Standard sample. (B) MG63 cells treatment with TW80-SeNPs and TW80-SeNPs + met examined by qPCR. The internal reference gene was GAPDH. The RNA extraction solution of (a) MG63 cells; (b) MG63 cells treatment with TW80-SeNPs; (c) MG63 cells treatment with TW80-SeNPs + met.

We then systematically studied the metabolism of TW80-SeNPs and analyzed metabolites SeIV, SeVI, SeCys_2_, MeSeCys, and SeMet in MG63 cells after 12 h and 24 h treatment by HPLC-ICP-MS. It can be seen that Se can hardly be detected in the cell protein extract in control group (Figure 6A). However, MG63 cells treated with TW80-SeNPs and TW80-SeNPs + met for 12 h showed high levels of SeIV and trace MeSeCys and SeMet, and the content in the TW80-SeNPs + met group was more than that of TW80-SeNPs group. After treatment for 24 h, the Se metabolites of MG63 cells showed SeIV and trace SeMet and SeVI. Moreover, the SeIV, MeSeCys and SeMet content in the 12 h group was higher than that in the 24 h group.

In addition, considering the important biological roles of Se existed in numerous proteins, we further determined the transcript abundance of selenoproteins of cells treated with TW80-SeNPs by qPCR [49]. The expressions of SeII, SeIR, SeIS, SeIW, GPx1/2, and DOI 1/2 showed different trends at 3 h and 6 h in the combined group (Figure 6B). It is confirmed that the combination of drugs can significantly reduce the expression of SeIH, SeIM, SeIO, and SeIP, and enhance the expression of GPx4, TR1, and Sep15 (Figure 6B). GPx4 and TR1 play an important role in maintaining intracellular redox balance [50–52]. Therefore, we speculate that the metabolism of TW80-SeNPs into selenoproteins enhances the anticancer effect of NK92 cells by regulating the redox balance in the tumor microenvironment.

## 4 Conclusions

From the anticancer effect of functionalized SeNPs and met combined with NK92 cells in HepG2 and MG63 cells, it was found that functionalized SeNPs could enhance the immunotherapy effect of NK92 cells. Among them, TW80-SeNPs combined with met had the best enhancement effect on NK92 cells. The main mechanism of action is that TW80-SeNPs combined with met may increase the level of ROS in HepG2 cells and the expression levels of cell surface receptor proteins ULBP-3/4, PD-L1, MICA and NK92 cell surface receptor proteins PD-1 and FasL. On the other hand, we found that after TW80-SeNPs was absorbed by MG63 cells and internalized into cells, it was gradually metabolized into SeIV, which regulated various selenoproteins in cancer cells, reduced the expression levels of SeIH, SeIM, SeIO, and SeIP, and increased the expression levels of Gpx4, TR1, and Sep15. The study of functional SeNPs combined with met and NK92 cells in tumor therapy is expected to provide a therapeutic strategy in treating tumor and diabetic patients complicated with cancer, and provide an idea for multidrug combination to enhance immune cell function and the application of immunotherapy.

## Supplementary Material

Supplementary Material Details
