# Supplementary material for: Highly active selenium nanotherapeutics combined with metformin to achieve synergistic sensitizing effect on NK cells for osteosarcoma therapy
Source: Nanophotonics. 2022 Jun 23;11(22):5101–11. doi: 10.1515/nanoph-2022-0289 (PMC11501141; doi:10.1515/nanoph-2022-0289)
Supplement: Supplementary file 1 — Supplementary Material Details [file j_nanoph-2022-0289_suppl.docx]

**Supporting information**

**Highly active selenium nanotherapeutics combined with metformin to achieve synergistic sensitizing effect on NK cells for cancer therapy**

Yanxin Du^1^, Zehang Zhang^2^, Yu Yang^2^, Ting Liu*^2^, Tianfeng Chen^2^, Xiaoling Li *^3^

^1^ The Second Clinical Medical College, Guangzhou University of Chinese Medicine, Guangdong Provincial Hospital of Chinese Medicine, Guangzhou, China;

^2^ Department of Oncology, The First Affiliated Hospital, Department of Chemistry, Jinan University, Guangzhou 510632, China;

^3^ Institute of Food Safety and Nutrition, Jinan University, Guangzhou 510632, China.

*Corresponding author.

Dr. Ting Liu, Email: liut_8800@jnu.edu.cn.

Dr. Xiaoling Li, Email: tlxlli@jnu.edu.cn.

**Results**

**Figure S1 Cell viability of functionalized SeNPs or Met in HepG2 cells.** The cell viability of **(A)** functionalized SeNPs, **(B)** Met in HepG2 cells for 72 h. Each value represents as means ± SD (n=3).

**Figure S2 Cell viability of functionalized SeNPs and Met for NK92 cells.** The cell viability of **(A)** PAH-SeNPs, **(B)** PVP-SeNPs, **(C)** TW80-SeNPs, **(D)** Met in NK92 cells for 24 h. Each value represents as means ± SD (n=3), **p*＜ 0.05, ** *p*＜ 0.01, *** *p*＜ 0.001.

**Figure S3** **Expression levels of receptor signaling molecule on HepG2 cells and NK92 cells after treatment with TW80-SeNPs combined with Met for 24 h. (A)** ULBP1, **(B)** ULBP2 and **(C)** PD-L1, **(D)** the MFI value of each receptor signaling molecule on HepG2 cells. **(E)** PD1, **(F)** the MFI value of each receptor signaling molecule on NK92 cells. Each value represents as means ± SD (n=3), **p*＜ 0.05, ** *p*＜ 0.01, *** *p*＜ 0.001.

**Figure S4 ROS levels of HepG2 cells after treatment with TW80-SeNPs combined with Met for 10 h.** Each value represents as means ± SD (n=3), **p*＜ 0.05, ** *p*＜ 0.01, *** *p*＜ 0.001.
